# The impact of rapid weight regain groupings on fight outcomes in professional mixed martial arts

**DOI:** 10.1080/15502783.2025.2550159

**Published:** 2025-08-25

**Authors:** Arjun Mote, Gabriel J. Sanders, Cassandra Evans, Corey A. Peacock, Jose Antonio

**Affiliations:** a Nova Southeastern University, Department of Biological Sciences, Davie, FL, USA; b University of Cincinnati, Exercise Science, Cincinnati, OH, USA; c Nova Southeastern University, Department of Health and Human Performance, Davie, FL, USA

**Keywords:** Weight regain, victory, fight style, MMA

## Abstract

**Background:**

Rapid weight regain (RWRG) following official weigh-ins is a common practice among professional Mixed Martial Arts (MMA) athletes. While prior studies have examined weight regain across weight classes, few have explored outcomes based on percentage groupings of regained weight. Understanding whether RWRG affects fight performance or method of victory may offer valuable insight for athlete preparation and regulatory policy.

**Methods:**

A total of 308 professional MMA bouts were analyzed using public records. All weigh-ins were supervised by the California State Athletic Commission (CSAC) with a commission-calibrated scale. Fighters were categorized into five groups based on the percentage of weight regained between weigh-in and competition: <3% (Low), 3–6% (Low-Moderate), 6–9% (Moderate), 9–12% (Moderate-High), and >12% (High). Chi-square tests assessed the association between weight regain group and both overall fight outcome (win vs. loss) and method of victory (KO/TKO, submission, decision). Statistical significance was set at *p* ≤ 0.05.

**Results:**

Chi-square analysis revealed a significant association between weight regain group and method of victory (χ^2^ = 16.345, *p* = 0.038). Fighters who regained a higher percentage of body weight were more likely to win by KO/TKO, while those with minimal regain tended to win by decision. No significant association was found between weight regain group and overall fight outcome (χ^2^ = 8.070, *p* = 0.089). No other significant results were observed (*p* ≥ 0.05).

**Conclusion:**

Categorizing MMA athletes by rapid weight regain percentage highlights differences in how fights are won, but not who wins. Fighters regaining a higher percentage of weight were more likely to win by KO/TKO, while those with minimal regain more often won by decision. These findings suggest that weight regain may influence fight style or pace but does not predict victory. Fight outcomes likely depend on other factors such as technique, training, and mental preparation. Future research should examine the physiological effects of RWRG and its implications for performance, policy, and athlete health.

